# Environmental harshness does not affect the propensity for social learning in great tits, *Parus major*

**DOI:** 10.1007/s10071-024-01862-w

**Published:** 2024-03-12

**Authors:** Emil Isaksson, Julie Morand-Ferron, Alexis Chaine

**Affiliations:** 1 Department of Biology, University of Ottawa, Ottawa, K1N 6N5 Canada; 2 Station d’Ecologie Théorique et Expérimentale du CNRS UAR2029, 2 route du cnrs, 09200 Moulis, France; 3 Institute for Advanced Study in Toulouse, 21 alleé de Brienne, 31015 Toulouse, France

**Keywords:** Social information, Individual differences, Cognitive ecology, Altitudinal differences, Social behaviour

## Abstract

According to the harsh environment hypothesis, natural selection should favour cognitive mechanisms to overcome environmental challenges. Tests of this hypothesis to date have largely focused on asocial learning and memory, thus failing to account for the spread of information via social means. Tests in specialized food-hoarding birds have shown strong support for the effects of environmental harshness on both asocial and social learning. Whether the hypothesis applies to non-specialist foraging species remains largely unexplored. We evaluated the relative importance of social learning across a known harshness gradient by testing generalist great tits, *Parus major,* from high (harsh)- and low (mild)-elevation populations in two social learning tasks. We showed that individuals use social learning to find food in both colour-associative and spatial foraging tasks and that individuals differed consistently in their use of social learning. However, we did not detect a difference in the use or speed of implementing socially observed information across the elevational gradient. Our results do not support predictions of the harsh environment hypothesis suggesting that context-dependent costs and benefits as well as plasticity in the use of social information may play an important role in the use of social learning across environments. Finally, this study adds to the accumulating evidence that the harsh environment hypothesis appears to have more pronounced effects on specialists compared to generalist species.

**Supplementary Information:**

The online version contains supplementary material available at 10.1007/s10071-024-01862-w.

## Background

Survival is greatly impacted by the spatiotemporal variation in resource availability, and some environments present greater challenges than others. Cognitive adaptations, which concern how animals collect, store, and use information (Shettleworth 2010), are one means of coping with food scarcity. For example, heightened spatial learning and memory evolved for storing food for later use (Sherry 1989). The “harsh environment” hypothesis (henceforth HEH) for the evolution of cognition proposes that environments characterized by uncertain access to food due to climatic variables, such as temperature and precipitation, should favour cognitive abilities that help mitigate this uncertainty (Pravosudov and Clayton 2002; Roth et al. 2010; Tebbich and Teschke 2014; Hermer et al. 2018). Altitudinal and latitudinal gradients have been frequently used to test the HEH, as increases in either correlate with colder, longer, and more intense (i.e. harsher) winters which restrict access to food and exert higher metabolic demand on endothermic animals (Pravosudov and Clayton 2002; Freas et al. 2012; Roth et al. 2012; Heinen et al. 2021).

The HEH has been repeatedly tested in scatter-hoarding species, such as black-capped (*Poecile atricapillus*) and mountain chickadees (*P. gambeli*), that rely on spatial memory and learning to retrieve food that they have hidden throughout their home range (Pravosudov and Clayton 2002; Freas et al. 2012; Tello-Ramos et al. 2018). Outside of such food-hoarding systems, the wider applicability of the HEH is still largely unknown. Indeed, non-hoarding species likely experience selection pressures on different cognitive abilities than hoarding species that rely primarily on spatial memory. However, the limited number of studies on non-hoarding species show mixed results. For example, woodpecker finches (*Cactospiza pallida*)*,* from an arid (harsh) area, were faster at reversal learning than individuals from a cloud forest (mild), which is consistent with the HEH (Tebbich and Teschke 2014). In contrast, two studies of great tits showed no support for the HEH. Great tits (*Parus major)* from mild- and low-elevation environments were more accurate than individuals from harsh, high-elevation environments in a serial reversal learning test (i.e. learning flexibility, Hermer et al. 2018). Furthermore, great tits from high and low elevations showed no difference in the accuracy of short-term spatial memory (Hermer et al. 2021).

Studies of the HEH have largely focused on individual “trial-and-error” learning in the absence of social influences, known as *asocial learning* (Heyes 1994), despite the widespread use of social learning in animals (Galef 1976; Galef and Laland 2005; Hoppitt and Laland 2008; Aplin 2019). Compared to asocial learning, a social learner learns from the successes and failures of others (Galef 1988; Shettleworth 2010), which allows them to gain knowledge about the environment with lower risks and costs in terms of time and energy spent (Boyd and Richerson 1988; Galef and Giraldeau 2001; Griffin 2004). However, social learning can also provide less accurate information as it is not first-hand information, potentially leading to suboptimal behaviour (Giraldeau et al. 2002). The balance of these costs and benefits also likely depends on the environmental context. Furthermore, social interactions within groups create unequal access to social learning opportunities depending on each individual’s social phenotype (Coussi–Korbel and Fragaszy 1995; Lonsdorf and Bonnie 2010). This can lead to differences in the propensity to use social information among individuals within a population (Aplin and Morand-Ferron 2017). Social learning might thus be consistent within individuals but vary with shifts in environmental states.

Despite the benefits of social learning when an individual has limited or incomplete personal information, there is theoretical debate as to whether social learning provides an advantage in uncertain or harsh environments leading to two contrasting views. On the one hand, the suggestion that social learning can provide sub-optimal information about the environment (Giraldeau et al. 2002), combined with increased severity of consequences for erroneous decisions in harsher environments, has led to the theory that asocial learning should be favoured over social learning in variable environments (Boyd and Richerson 1988; Laland and Kendal 2003; Aoki and Feldman 2014). On the other hand, recent theoretical models show that social learning could be favoured in variable environments (Rendell et al. 2010; Arbilly et al. 2011) as it could reduce the costs of foraging. Empirical tests of whether social learning is favoured with increased environmental uncertainty or not are rare. Mountain chickadees from harsh, high-elevation use social learning, but less than conspecifics at low elevation, consistent with the first theory (Heinen et al. 2021, 2022). Mountain chickadees are food-hoarding specialists that rely on asocial learning and memory to create and retrieve food caches for winter survival, so lower use of social learning and higher use of asocial learning at high elevation is consistent with predictions from the HEH. Whether the HEH holds for foraging generalists that could greatly benefit from social information about alternative food sources unlike specialist species remains unknown.

In this study, we compared the use of social learning in wild great tits from populations located at high (800–900 m asl) and low (400–500 m asl) elevations to assess how the HEH applies to social learning in a generalist species. Great tits are a common European foraging generalist that is related to chickadees and known to use social learning to access a variety of food sources (Fisher and Hinde 1949; Aplin et al. 2013; Brodin and Urhan 2014). Birds from these two elevations brought into captivity have been used to show that low-elevation great tits have higher reversal learning performance than high-elevation great tits (Hermer et al. 2018). The elevational contrast is marked by differences in temperature (~ 8 °C; Bründl et al. 2020) and snowpack which should increase variability in food availability and impact the importance of food for survival. Furthermore, breeding behaviour is delayed and breeding success is lower at high elevations in the region for both the great tit (Sallé 2016) and the closely related blue tit, *Cyanistes caeruleus* (Lejeune et al. 2019; Bründl et al. 2020). Here, we designed two foraging tasks and trained great tits to act as demonstrators for naïve observers for each task. We were specifically interested in the onset of socially learnt behaviours in the observers and thus took steps to exclude influences of asocial learning. We first asked if individuals use social learning in colour-associative and spatial learning foraging tasks and if the propensity to use social information differed across environments (elevation) both in terms of (1) approaching the task and (2) solving the task. If the theory that asocial learning is preferred over social learning in uncertain environments holds, we predict that behaviours associated with social learning should decrease as elevation increases. Conversely, if social learning is preferred over asocial learning in unpredictable environments, as suggested in recent models, then we predict that behaviours associated with social learning should increase as elevation increases. Second, we examined if there was evidence for a social learning phenotype in these populations by asking if individuals consistently differed in their use of social learning use across colour-associative and spatial tasks through repeatability analysis.

## Materials and methods

### Capture and handling

**Fig. 1:**
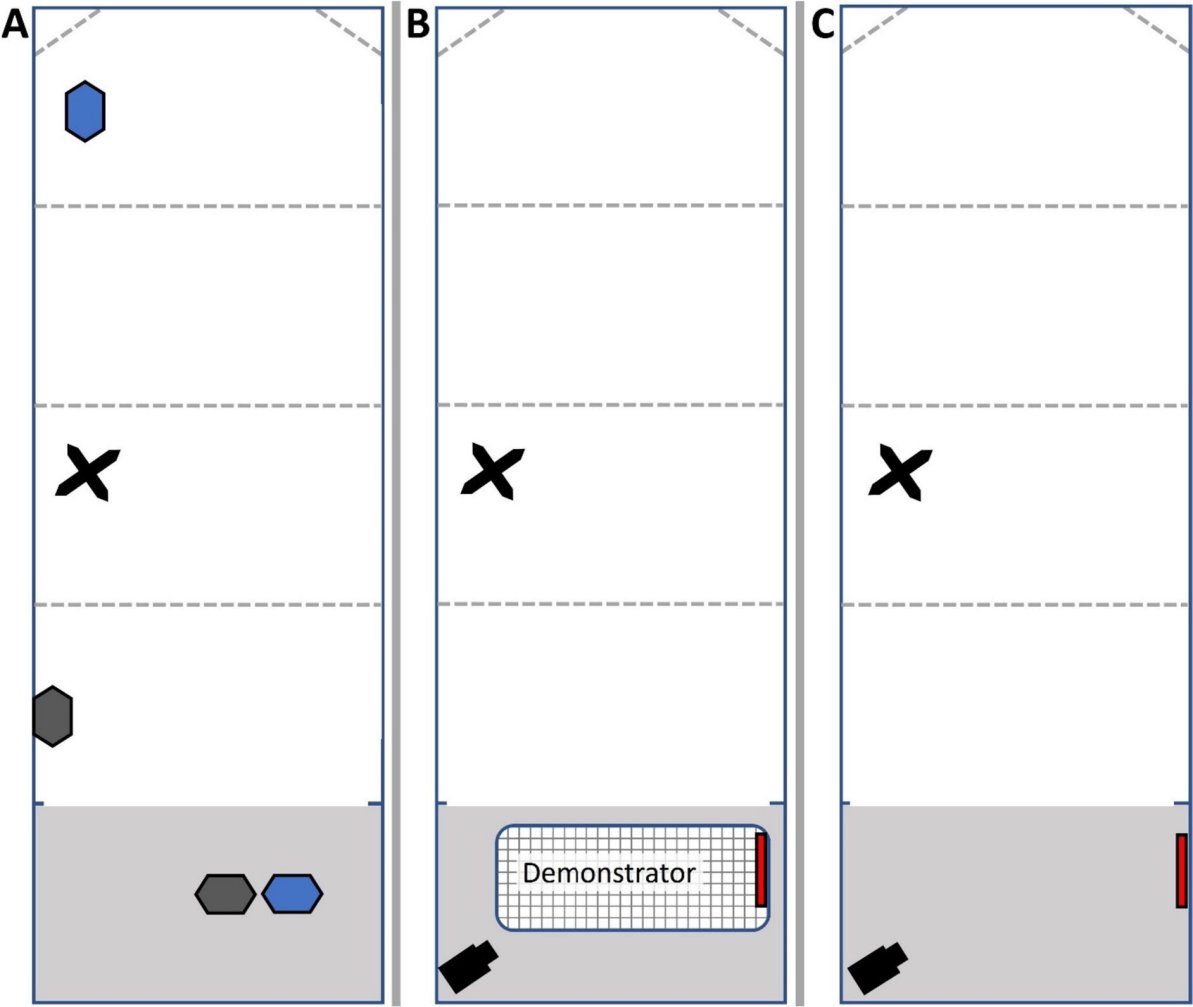
Top-down view of an observer aviary across the three experimental stages, **A** Pre- and post-social learning experiment, **B** during demonstration, and **C** during observer testing. The light grey and white sections indicate the indoor and outdoor compartments, respectively. The dotted lines indicate perches, and the black X mark represents an artificial tree also used as a perch. Blue and grey hexagons in panel **A** represent locations for food and water. The black camera silhouette in the lower left corner of panels **B** and **C** indicates camera placements. The hatch-marked rectangle labelled “Demonstrator” in panel **B** indicates the placement of the demonstrator cage with the smaller, red rectangle indicating the placement of the demonstrator board. The red rectangle in panel **C** indicates the placement of the test board

We captured great tits from Oct 2019 to Mar 2020 (three batches) and Oct 2020 to Dec 2020 (two batches) in the French Pyrenees using mist nets at high (800–900 m asl; *n* = 37) and low (400–500 m asl; *n* = 37) elevation sites. This elevation difference has been linked to environmental harshness having negative effects on life history traits in great and blue tits (Sallé 2016; Lejeune et al. 2019; Bründl et al. 2020), as well as differences in asocial learning flexibility (Hermer et al. 2018). We used three high (Antras: 42.88111″N 0.94563″E, Cap de Sour: 42.93118″N 1.12235″E, Villargein: 42.90389″N 1.04648″ E) and two low (Aubert: 42.96429″N 1.10310″E, Montjoie: 42.99886″N 1.16945″E) elevation sites. Following capture, birds were taken to the Station d’Ecologie Théorique et Expérimentale (SETE; 430 m asl) field station in Moulis where birds were banded, aged, and sexed following standard procedures (Svensson 1992). Birds were designated either as *observers* (*n* = 20 per elevation), *controls* (*n* = 7 per elevation), or *demonstrators* (10 adult males per elevation) and released into individual aviaries at the field station (Fig. 1A). We were unable to balance age and sex within and across elevations due to constraints imposed by capture methods, availability of birds, and aviary space. Likewise, we chose to increase the number of observers relative to controls given the limited number of birds and aviaries available. Each batch of birds was released back into the wild before the next batch was captured (sample composition details in supplementary material S1).

### Housing

Observers were housed in individual aviaries that had both outdoor (*W* = 1 m, *L* = 4 m, *H* = 3 m) and indoor compartments (*W* = 1 m, *L* = 1 m, *H* = 2.5 m) for shelter and experiments (Fig. 1). Birds were separated by one empty aviary such that they could see and hear each other (i.e. social enrichment) while in the outdoor compartment, but would not be able to observe social learning trials of their neighbours, which were conducted in the indoor compartments (Fig. 1). Outdoor compartments contained natural vegetation and several perches. Ad libitum food (mealworms, *Tenebrio molitor*, sunflower seeds, and suet cake) was provided in both compartments before and after experimental tests (Fig. 1A). Indoor compartments had a roosting box and a heater and light that were on for the first two nights to ensure acclimation to the aviary. Birds were held for a total of 30 days for other experiments, and social learning tests were conducted between days 16 and 25 of captivity.

### Demonstrators and demonstrator housing

Only adult male great tits were chosen as demonstrators to reduce variance among demonstrators (e.g. Aplin et al. 2015; Hämäläinen et al. 2020). Demonstrator individuals were housed and trained in separate mobile cages (internal dimensions: *W* = 0.58 m, *L* = 0.355 m, *H* = 1.13 m), which were all contained within one large indoor aviary for a given batch (four cages for each session) to provide social enrichment, except for batch 1 in which the demonstrators were kept in separate, smaller indoor aviaries. Demonstrator cages contained several perches, natural vegetation, ad libitum food (sunflower seeds, mealworms, and suet cake), and water. Demonstrators were habituated to task components prior to training by mounting an empty task board (see below) on a wall in the cage and hanging a bowl with five differently coloured cotton balls and two artificial leaves used in the learning tasks next to the board. Demonstrator birds were trained in either the *Colour* or the *Spatial* task (see below).

### Learning tasks

**Fig. 2:**
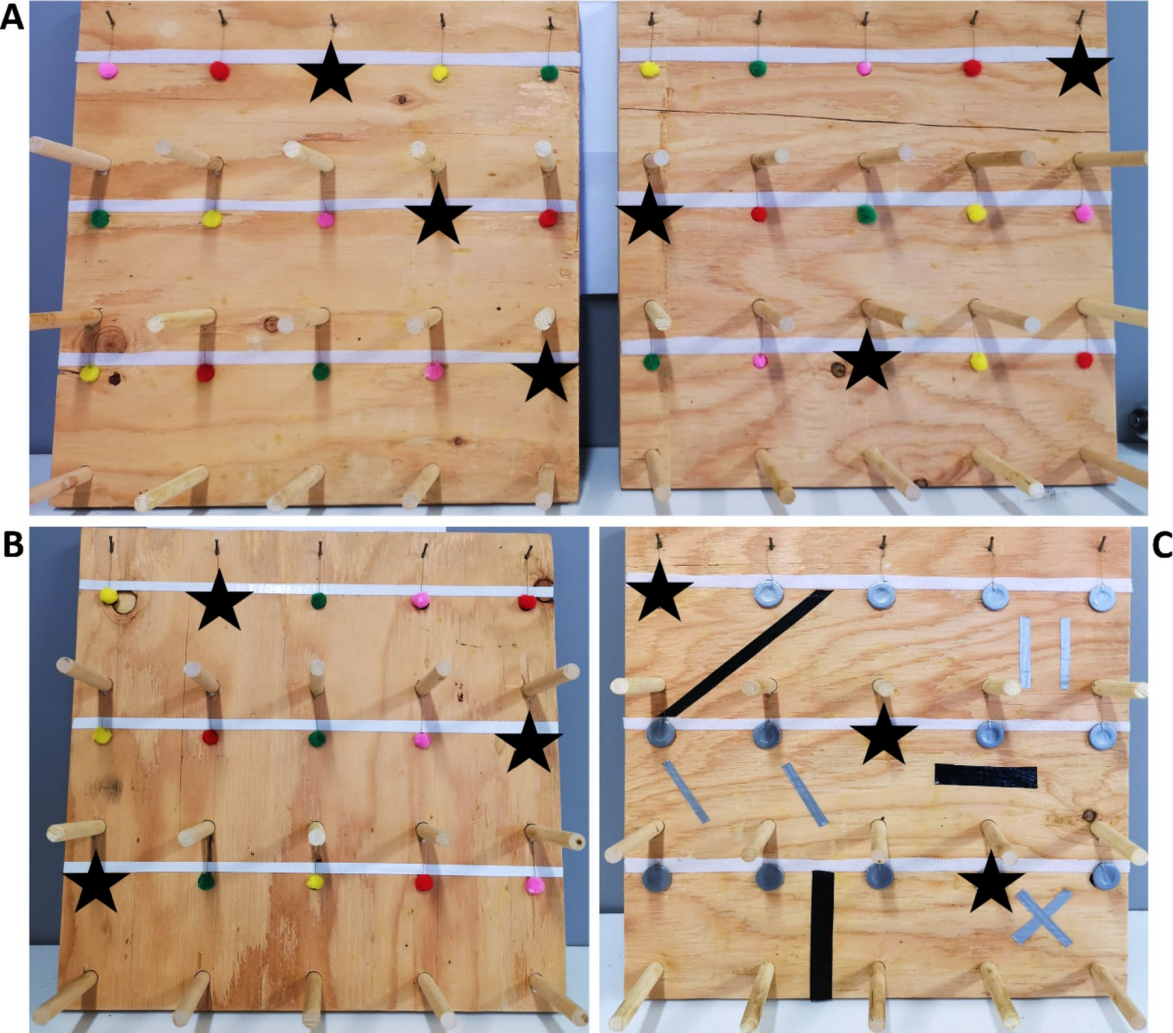
Training and testing board schematics. **A** The two configurations of coloured pompoms used to train the demonstrators and used during demonstrations in the Colour task. **B** The testing board used when testing observers in the Colour task. **C** Design and configuration of the Spatial task, both during training and observer testing, including the landmark patterns in grey and black tape. Black stars indicate the correct choices (blue pompom in the Colour task, and artificial leaves in the Spatial task), both on the training boards (panels **A** and **C**) and the testing boards (panels **B** and **C**)

Each observer bird (*n* = 40) was tested for social learning on both learning tasks, performed by demonstrator birds, on separate days. The colour discrimination learning task (*Colour*) and the spatial learning task (*Spatial*) aimed to engage different contexts of the cognitive process for the birds (what vs. where). Both tasks used wooden testing boards (32W x 32Hx 2D cm) with 15 evenly spaced wells (Fig. 2), and a perch in front of each well that the birds could use to easily inspect the well. During demonstrator training, a mealworm was placed in specific wells as a reward for correct choices. In the *Colour* task, observers were tested on their ability to learn to sample blue cotton balls, called “pompoms” (Hobbyworld Arts and Crafts Inc.), from an array including four other coloured pompoms (green, yellow, pink, red) used to cover the feeding wells (Fig. 2A). *Colour* task boards had an equal number of each pompom colour (three each). In the *Spatial* task, observers were tested on their ability to learn three locations on a testing board that included spatial landmarks (patterns made with grey and black tape), and on which all wells were covered by grey, artificial “leaves” (washers covered in tape, Fig. 2C). Pompoms and “leaves” were attached to the boards with fishing lines so that they returned to their original position covering the well again following sampling. Pompoms and “leaves” have been successfully used previously in avian cognition research (Roth et al. 2010; Rojas‐Ferrer et al. 2020).

### Demonstrator training

All habituation items and food other than rewards on the learning board were removed during training and demonstration sessions. Two demonstrator individuals in each batch were trained in the *Colour* task, and the other two were trained in the *Spatial* task (one high- and one low-elevation bird for each task). Training followed a successive approximation methodology in which the birds were presented with wells fully uncovered first, and for each training step that the bird passed, the wells were covered more until wells were completely covered. Rewarded wells (Fig. 2) contained immobilized (decapitated) mealworms. This type of training method has been used successfully in previous experiments with related species (Thompson and Morand-Ferron 2019; Rojas‐Ferrer et al. 2020). All demonstrator birds in our experiment successfully learned their task prior to social learning experiments.

Demonstrators trained in the *Colour* task were trained over four steps. Each *Colour* task demonstrator was trained with two different colour configurations on the board (Fig. 2A), to ensure that demonstrators learned the colour association rather than the spatial location of rewarded wells. Each training step was repeated at least twice per training board, except step 1 (fully uncovered wells) for board 2, and the demonstrator had to choose the three rewarding items first in consecutive repeats to move to the next step. In the last step of training (fully covered wells), a demonstrator had to choose the three blue pompoms first across twelve consecutive presentations, alternating training boards 1 and 2 every two presentations, with a maximum of three errors across all twelve presentations to be considered trained.

Demonstrators for the *Spatial* task were trained over five steps starting with no leaf in front of the wells and ending with the leaf fully covering the wells. Demonstrators were trained on identical boards to those used to test observers in the spatial task (Fig. 2C). Each training step was repeated at least three times, and a demonstrator was considered fully trained in the task when they successfully pulled the correct leaves first when presented with a board with all wells covered and did so over five consecutive repetitions. Although we did not allow any sampling errors for reaching this criterion, we did not consider quick pecks on the outer face of a leaf by the demonstrator as a sampling error since these differed considerably from attempts to get behind the leaf. A sampling attempt was defined as an interaction with a leaf that lasted at least 1 s involving manipulation of the leaf to get behind it (pulling, lifting, or pecking from the side so that the leaf lifted from the board).

### Individual activity level

Consistently, more active individuals could have a higher chance of approaching and interacting with the test boards by chance alone, so we estimated the mean activity level of each observer and control bird before social learning tests. Over 3 days preceding the social learning test, we recorded the number of continuous movements longer than one body length made by birds during a three-minute period in the afternoon. Individuals in batches 1 and 4 were only scored on two rather than 3 days due to time constraints, resulting in a total of 148 scoring sessions across all 40 individuals.

### Social learning procedure and variables

All observer birds were assayed first on the *Colour* task over 2–3 days, with a maximum of three observers assayed per day, and then assayed in the same order in the *Spatial* task over 2–3 days to keep the time between tasks consistent among birds. The order of task presentation was kept the same throughout the study to avoid introducing variance in observer performance that might exist if the chance of success in one task impacts the chance of success in the second task differently depending on which task was presented first. 2 days before the first social learning presentation, habituation items were placed inside the observer’s aviary to minimize item neophobia during social learning tests. These included an empty testing board without pompoms, leaves, or markings, mounted 1.5 m off the ground on one side of the indoor chamber (same location as during observer testing, Fig. 1C), and a bowl containing one pompom of each colour and two artificial leaves. Habituation items and food, including food dropped on the floor by the bird, were removed from the focal observer’s aviary one hour before social learning tests began. During this food deprivation period, two stacked video cameras (Sony HD Handycam) were placed in the aviary so that the observer could acclimatize to them. After experimental testing, food and habituation items were placed back in the aviary until the second social learning test.

Tests were conducted between 09.00 and 18.00, with no individual starting their test after ~ 16:30 to minimize the effects of inactivity after sunset (except for one individual). Demonstrators were food deprived for 30–45 min prior to tests to ensure they would demonstrate the learning task during the trial. During social learning experiments, a total of four demonstrators did not perform their trained task within the first 15 min of the demonstration and were not used for subsequent tests (see below). Observer and control bird motivation was verified before the first trial of each task by providing a single mealworm in the regular feeding place at the entrance to the indoor aviary section, and the observer had to consume it before tests began. If they did not eat the mealworm, we tried again after a five-minute break and continued this pattern until the mealworm was consumed. Therefore, all observer and control individuals were food-motivated at the start of social learning trials.

Once motivation was verified, the door separating the indoor and outdoor compartment of the aviary was closed and the demonstrator cage was placed in the observer’s aviary (Fig. 1B). A task-appropriate demonstration board with mealworms behind the trained colours or locations was then placed in the demonstrator cage (Fig. 1B). Finally, the door between the compartments was then opened and the experiment began. The demonstration ended when either all three worms were taken or when the 15-min time limit was reached. An observer’s social learning assay consisted of a maximum of five consecutive trials where each trial included a demonstration followed by an observer test. Each demonstration provided the observer with up to three opportunities to learn to approach and sample the correct items (the three rewarded wells). Demonstrators extracted worms from behind all three correct items in most (94%) trials and from behind two items or one item in 5% and 1% of trials, respectively. If a demonstrator failed to reveal any worms, a five-minute break was given before a new attempt. During this break period, the demonstrator was left in the observer aviary but the door between the indoor and outdoor compartments was closed. In one case, the demonstrator failed a second time and it was replaced by a new demonstrator. If a demonstrator extracted fewer than three worms across five trials for a given observer, we deemed it a poor demonstrator and it was replaced by another demonstrator for the remaining observer tests that day. By testing observers in their long (4 m) home aviary, it is possible that some observers might not closely watch the demonstrator. Individuals who are not interested in social learning are not expected to pay attention to demonstrations and allowing birds the opportunity to pay attention to demonstrations or not closely resemble the natural context of social learning responses from observers. However, there are three reasons to believe that most birds noticed demonstrators in our trials. First, food and tasks for other experiments were provided in the indoor space where the observer was placed, so birds generally were attentive to that space (Fig. 1A). Second, the cages were small enough that it would be impossible for the bird to not notice the presence of a demonstrator. Finally, birds with a demonstrator showed differences in the behaviour from those who did not get a demonstrator (see results below), suggesting that in most cases observers paid attention to the presence of the demonstrator.

Following a demonstration, the demonstrator cage was removed from the observer’s aviary. The observer’s testing board without mealworms was then mounted on the wall of the indoor compartment in the same location that the demonstrator’s board had been, and the five-minute trial began. The board was empty of mealworms as we specifically wanted to separate the effects of social information generated by the demonstrator from other cues such as the smell or sound of a mealworm on the board. During this period, we watched the observer through a viewing window and recorded: (1) whether the bird perched on/near (within one body length) of the test board, (2) whether a bird sampled an item on the board and how many items it sampled, (3) latency until first perch on/near the test board, and (4) latency until sampling both the first well overall and first correct well (the first correct well could also be the first overall). EI scored behaviours for all trials. EI conducted direct observations of all observer trials and most control trials and scored videos of all control trials conducted by assistants. Video recordings were used to verify the accuracy of events in trials that were scored live for a subset of tests. Behaviours scored were clearly defined (perching, pushing/pulling pompoms, or leaves), and behaviours scored from video universally matched live scored measures. Latencies were calculated as the cumulative number of seconds across trials until the observer interacted (perched/sampled) with the board. If the observer did not sample any items during the 5-min trial, a new trial was given (demonstration and test). Each trial was a maximum of 301 s (five minutes + 1 s), with a maximum total of 1505 s across five trials. One second was arbitrarily added to each trial without the bird interacting with the board to avoid potential instances when an observer would perch/sample at exactly 1500 s. If the observer sampled any items in a trial, it was not tested again to avoid the influence of direct asocial learning on performance in subsequent trials. This point is important as it excludes the negative feedback of sampling a correct answer that did not contain a worm from the response we scored.

### Control

To verify that the actions of the observer were influenced by the actions of the demonstrator rather than asocial learning, we conducted control tests on seven individuals from each elevation (composition in supplementary material S1). These control tests used the same methods as the social learning tests but without demonstration sessions. We chose to not present a bird, rather than an untrained bird, for these control tests since the presence of another bird in their cage could contribute to social information leading to local enhancement (approaching a foraging board). Local enhancement refers to when the likelihood of an observer visiting a location increases following a demonstration (Thorpe 1963; Shettleworth 2010). By excluding any social information available in the control tests, we were able to quantify the social influence on observer behaviour. Birds were tested for five minutes with a five-minute break between trials during which the test board was removed from the aviary. As for the observer birds, trials continued until a bird sampled a well in a trial or failed in all five trials. The behaviour of the birds experiencing the control trials was scored the same way as observer birds.

### Statistical analysis

Data handling and statistics were conducted in R [version 4.2.1] (R Core Team 2020). To consider the inclusion of individual activity levels as a covariate in social learning analyses, we first evaluated the reliability of our measure of individual activity level. We evaluated individual repeatability in activity level using the *rptR* package [0.9.22] (Stoffel et al. 2017). We estimated the repeatability of activity level for individuals measured more than once (Bird ID) while controlling for time of day as a fixed effect and the time of year (Batch) and the experimenter who recorded the measure (Person) as random effects. We ran 1000 bootstrap resampling (*n* = 53, missing activity score for one individual) to estimate the significance of each random effect using a likelihood ratio test. Based on the results from the repeatability test (see Results), mean individual activity level (2–3 days; see above) was added as a continuous variable, mean centred and scaled, for all relevant analyses.

We tested all controls and observers on two aspects of social learning in each task. First, local enhancement is defined as whether the birds perched on/near the test board (Yes/No). Second, action copying was scored for whether a bird sampled the test board by interacting with an item (Yes/No, henceforth “action copying (Y/N)”) as well as the number of items sampled per individual (henceforth “action copying (#)”). Action copying in this study was used to indicate that observers interacted with any item on the board following a demonstration. We were unable to evaluate individual accuracy of action copying due to too few correct responses by observers across tasks (Table 2), so we chose to focus on general action copying with any item in the analysis. Finally, we were unable to acquire standardized measures of local enhancement for one individual and action copying for three individuals (including the one missing the local enhancement score) due to time constraints (Table 2). These missing values are indicated as “NA” in the descriptions below.

### Degree of local enhancement and environmental harshness

We first assessed the impact of elevation on social learning by comparing the degree of local enhancement between individuals from high vs. low elevation. We used a binomial generalized linear mixed-effects model (GLMM) with local enhancement as the response and elevation (high/low), test (observer/control), task (colour/spatial), individual activity, number of days since the start of the season (Oct 1st each year), sex (male/female), and age (juvenile/adult) as fixed effects and bird ID as a random effect to account for individual differences in responses across tasks (*n* = 54 birds; observations = 105, 3 NA). To evaluate the influence of receiving a demonstration on observer response between elevations, we allowed for an interaction between elevation and test in the model. The number of days since the start of the season was mean-centred and scaled prior to analysis. Mixed-effects regression models were constructed using the *lme4* package [1.1–30] (Bates et al. 2015), with significance tests from the *lmerTest* package [3.1–3] (Kuznetsova et al. 2017), and model assumptions were validated using the *DHARMa* package [0.4.5] (Hartig 2021). Graphics pertaining to this analysis were created using the *ggplot2* package [3.3.6] (Wickham 2016).

### Latency of local enhancement and environmental harshness

We also analysed how early in a trial individuals from either elevation used local enhancement as individuals might not differ in the ability to use local enhancement between elevations but rather how soon they use it. We used a mixed-effects survival model, which allowed close examination of time steps until an event (perching = Yes), and accommodated observations with an unknown endpoint (perching = No after the maximum of five trials) (Therneau 2020). We tested latency until perching as a response against elevation, task, individual activity, and days since the start of the season, sex, and age as fixed effects, while accounting for bird ID as a random effect (*n* = 40 birds; observations = 79, 1 NA). The control group was excluded from the survival analysis because we were interested in differences in the speed of social learning use across elevations given a demonstration. The proportional hazard assumptions were tested using the *cox.zph* function in the *survival* package [3.3–1] (Therneau 2021). Multicollinearity in the survival models was tested using the variance inflation factor function (“*vif”*) in the *rms* package [6.3–0] (Harrell 2021). The assumptions of i) linearity between the predictors and log hazard, ii) influential observations, and iii) outliers were tested on a non-mixed-effects version of the model that accounted for the random effect of ID by including it as a “frailty” argument in the model (see Therneau et al. 2003). The results of the mixed-effect survival model and the frailty model were qualitatively the same and thus validate the use of the frailty model to test assumptions. Linearity was evaluated with a residual plot using the martingale residuals of the frailty model plotted against the continuous predictor variable (activity level). Survival curves were illustrated using the *survminer* package [0.4.9] (Kassambara et al. 2021).

### Degree of action copying and environmental harshness

We investigated whether observer propensity for action copying depended on elevation using the same approach as for local enhancement. Given the count structure of our action copying (#) response, we used a negative binomial GLMM to test action copying (#) against elevation, test, task, individual activity, and days since the start of the season, sex, and age as fixed effects, allowing for an interaction between elevation and test. We also accounted for bird ID as a random effect (*n* = 54 birds; observations = 103, 5 NA).

### Latency of action copying and environmental harshness

Finally, we also tested for differences in latency of action copying (Y/N) as individuals might differ in how fast they implement acquired information rather than in their ability to do so. As for local enhancement, we used a mixed-effects survival model including the same model parameters and tests of assumptions as used to test local enhancement (*n* = 40 birds; observations = 77, 3 NA).

### Individual consistency in social learning

The benefit of social information to an individual in the wild may depend on both their internal and external environments (Webster and Laland 2011; Jones et al. 2017). Individuals are thus expected to be consistent in their willingness to use social learning across similar contexts. We therefore tested contextual repeatability (Cauchoix et al. 2018) of social learning at the individual level across the *Colour* and *Spatial* tasks. We used a binomial repeatability test for local enhancement with task (*Colour*/*Spatial*), individual activity, elevation, and days since the start of the season as fixed effects with bird ID as a random effect (*n* = 40 birds; observations = 79, 1 NA). We tested for repeatability of action copying (#) using a Poisson-adjusted repeatability test with the same model structure as for the local enhancement repeatability test (*n* = 40 birds; observations = 77, 3 NA). Both repeatability tests were evaluated using 1000 bootstraps resampling. The control group was excluded from the repeatability tests as we were interested in the consistency of social learning use per se following a demonstration. Finally, we estimated the influence of elevation on the repeatability of social learning by running repeatability tests with only elevation as the fixed effect.

## Results

### Repeatability of activity

Consistent individual differences in activity could influence the behaviours we scored for social learning. We found significant repeatability of activity at the individual level (ID; Table 1) while controlling for time of day, potential effects of the person who recorded the measurement (Person; Table 1), and seasonal differences (Batch; Table 1).

**Table 1:** Repeatability test for individual activity level

Random effect	R	SE	95% CI	*p*
ID	0.14	0.09	[0,0.323]	0.04*
Person	0.074	0.092	[0,0.298]	0.1
Batch	0.094	0.082	[0,0.291]	0.06

^*^significant at the 0.05 level

*Random-effect* indicates the level tested for repeatability, *R* indicates the repeatability coefficient, *SE* is the standard error, *95% CI* is the 95% confidence interval, and *p* is the p-value

### Task engagement summary

Two and three individuals, out of fourteen, in the control group perched on the *Colour* and *Spatial* tasks, respectively. In the social observer group, 21/40 perched on the *Colour* task and 28/40 perched on the *Spatial* task (Table 2). No control individuals sampled any items on the test board, whereas 13/40 observers sampled in the *Colour* task, and 9/40 observers sampled in the *Spatial* task (Table 2).

**Table 2:** Engagement rates, as the number of individuals that exhibited each measured response

Group	Elevation	Total N	Task	Perched	Sampled any item	Sampled at least one correct item	Perching NAs^a^	Sampling NAs^a^
Observers	High	20	*Spatial*	15	9	1	–	–
			*Colour*	12	8^b^	1	–	1
	Low	20	*Spatial*	13	9	4	–	–
			*Colour*	9^c^	5^d^	2	1	2
Control	High	7	*Spatial*	1	0	0	–	–
			*Colour*	0	0	0	–	–
	Low	7	*Spatial*	2	0	0	–	–
			*Colour*	2	0	0	–	–

^a^NA = individuals that were removed from the sampling behaviour analysis due to missing observations caused by time constraint in batch 1

^b^Data for one female were missing due to incomplete trials (i.e. final N for “High” = 19)

^c^One male was removed due to an incomplete trial

^d^Data from two males were missing due to incomplete trials (i.e. final N for “Low” = 18)

### Local enhancement and environmental harshness

Birds from high and low elevation showed no significant difference in their propensity to use local enhancement (12/20 high-elevation birds and 9/20 low-elevation birds, Table 2; GLMM: Table 3). There was a significant difference between test groups such that observers were more likely to approach the test board than control individuals (Table 3). No effect of the interaction between elevation and test group was detected in the model (Table 3). Finally, we did not detect any effect of task type (*Colour* vs *Spatial*), individual activity level, days into season, sex, or age on the propensity to use local enhancement (Table 3).

**Table 3:** Parameter estimates for the models of local enhancement (binomial GLMM, *N* = 79) and action copying (negative binomial GLMM, *N* = 77)

	Local enhancement: binomial GLMM				Action copying: negative binomial GLMM			
*Predictors*	*Estimate*	*SE*	*z-value*	*p*	*Estimate*	*SE*	*z-value*	*p*
Intercept	− 4.66	1.90	− 2.45	**0.01**	− 21.06	8735.73	− 0.00	1.00
Elevation (low)	2.76	1.96	1.41	0.16	− 0.05	12,339.76	− 0.00	1.00
Test (observer)	5.06	1.86	2.72	**0.01**	20.86	8735.73	0.00	1.00
Task (spatial)	0.89	0.56	1.58	0.11	0.11	0.39	0.29	0.77
Activity	0.61	0.56	1.10	0.27	− 0.14	0.30	− 0.45	0.66
Days into season	− 0.20	0.40	− 0.49	0.62	-0.32	0.21	− 1.49	0.14
Sex (male)	− 0.36	0.78	− 0.47	0.64	0.15	0.42	0.36	0.72
Age (juvenile)	− 0.40	0.95	− 0.42	0.67	− 0.12	0.46	− 0.25	0.80
Elevation (low) * Test (observer)	− 3.68	2.11	− 1.75	0.08	− 0.10	12,339.76	− 0.00	1.00
Random effects								
Residual	1				1.67			
Observer ID	2.54				0.00			

**Fig. 3:**
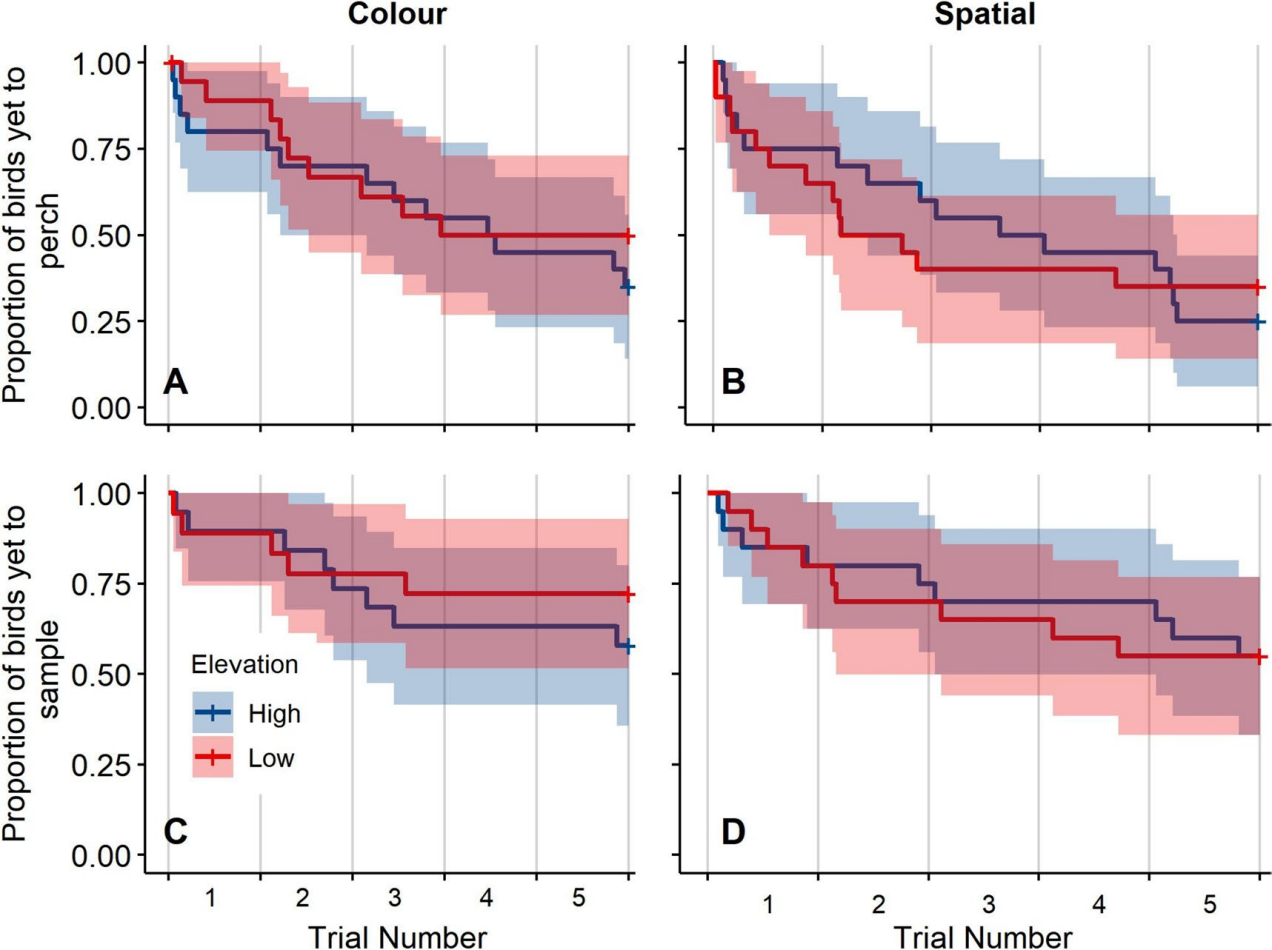
Survival plots of the proportion of individuals that engaged with the test boards over five trials for observer birds only (i.e. no control individuals). The graphs are based on the raw latency to engage with the boards plotted against elevation and not the full mixed-effects survival models. The proportion of individuals that engaged increases as the line decreases. The x-axis shows the time since the start of the first trial in seconds, and each tick represents the break between two trials. Blue and red lines represent high and low elevation, respectively, and the spread around the lines indicates 95% confidence intervals. Change in proportion of individuals that did not perch in the **A** Colour task (*n* = 80) and **B** Spatial task (*n* = 80) over time. Change in proportion of individuals that had not sampled any items from the test board in the **C** Colour task (*n* = 77) and D) Spatial task (*n* = 77) over time

Although individuals from different elevations did not differ in their propensity to use social information for local enhancement, they might differ in how quickly such information is used for local enhancement. In the mixed-effects survival model for latency to use local enhancement, 50 out of 79 possible perching events occurred, leaving 29 events right-censored (i.e. endpoint unknown). We found no significant difference in the latency of local enhancement between high- and low-elevation individuals (mixed-effects survival model: elevation (low) = − 0.05, *z* = − 0.11, *p* > 0.05, Table 4; Fig. 3A–B). Individual activity level had a significant effect on perching behaviour, with more active individuals being more likely to exhibit local enhancement (activity = 0.80, *z* = 2.17, *p* < 0.05, Table 4).

**Table 4:** Summary of the mixed-effects survival models

	Local enhancement					Action copying				
*Predictors*	*Log(hazard)*	*Hazard ratio*	*SE*	*z-value*	*p*	*Log(hazard)*	*Hazard ratio*	*SE*	*z-value*	*p*
Elevation (low)	− 0.05	0.95	0.49	− 0.11	0.92	− 0.15	0.86	0.53	− 0.29	0.77
Task (spatial)	0.42	1.53	0.31	1.37	0.17	0.24	1.27	0.38	0.64	0.52
Activity	0.80	2.22	0.37	2.17	**0.03**	0.30	1.35	0.36	0.83	0.40
Days into season	− 0.24	0.78	0.24	− 1.03	0.30	− 0.25	0.78	0.25	− 0.98	0.33
Sex (male)	0.18	1.20	0.48	0.38	0.70	0.18	1.20	0.51	0.35	0.72
Age (juvenile)	0.02	1.02	0.53	0.04	0.97	0.07	1.08	0.56	0.13	0.89

Log(hazard)  regression coefficient; a positive sign indicates a higher probability of perching/sampling. Hazard ratio effect size. *SE*  standard error

### Action copying and environmental harshness

**Fig. 4:**
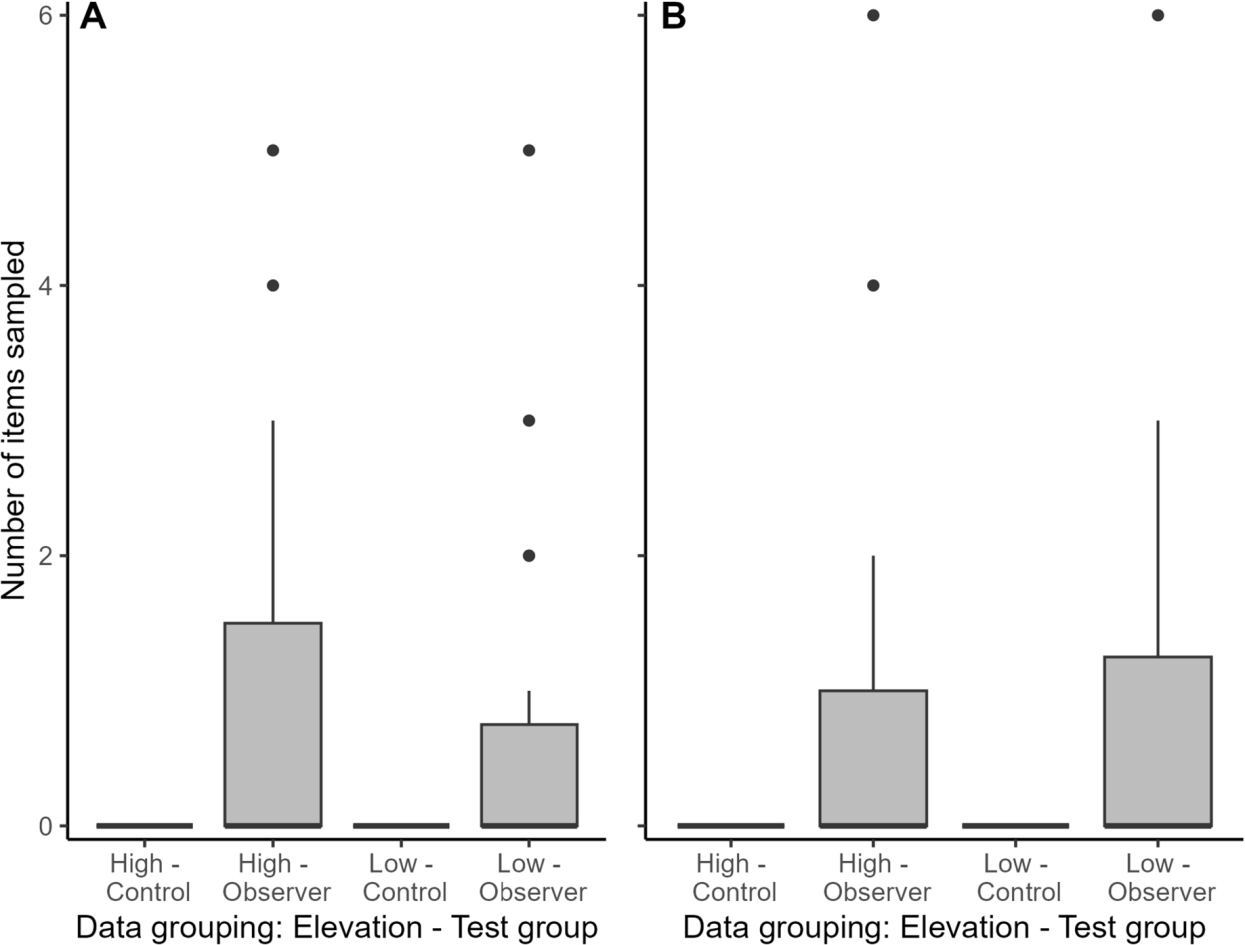
Number of items sampled (y-axis) in the Colour (**A**) and Spatial (**B**) tasks for each elevation and treatment group combination (x-axis). The thick black line represents the median, filled boxes indicate the 75th percentile, the lines indicate 1.5 IQR, and points show statistical outliers

High- and low-elevation populations did not differ significantly in their propensity for action copying across tasks (GLMM: *N* = 77 [3 observations missing], elevation (Low) = − 0.05, *z* = − 0.00, *p* > 0.05, Table 3, Fig. 4). Test, task, individual activity, days into season, sex, age, and the interaction between elevation and test group did not show a significant effect on action copying (Table 3). Finally, elevation did not significantly impact the speed of action copying, nor did any other variable included in the mixed-effects survival model (Table 4, Fig. 3C–D). The model detected 31 sampling events out of 77 possible events.

### Consistency in social learning

The individual consistency in the propensity to use social information across the colour-associative and spatial learning tasks could reveal the existence of a social learning phenotype. Individuals differed consistently from one another in their use of local enhancement across the two tasks (link-scale approximation: R = 0.40, SE = 0.25, 95% CI = [0, 0.99], *p* < 0.05). However, there was a negligible influence of elevation on consistency in local enhancement (partial R = 0.027 SE = 0.067). In contrast, the repeatability test showed that individuals did not differ consistently in their action copying behaviour across tasks (link-scale approximation: R = 0.04, SE = 0.118, 95% CI = [0, 0.40], *p* > 0.05). Elevation likewise did not influence the consistency of action copying (partial R = 0.003 SE = 0.038).

## Discussion

Social learning can reduce the costs of acquiring information about resources and should therefore be particularly important for survival in harsh environments where both the difficulty in finding resources and increased energetic needs are accentuated. Here, we investigated the use of social learning in populations of great tits, a generalist forager, across an elevational gradient known to affect life history traits in both great tits and the closely related blue tit in the study area (Sallé 2016; Lejeune et al. 2019; Bründl et al. 2020). We show that great tits that received demonstrations had higher success in our foraging-related tasks than individuals that did not receive a demonstration. However, there was no difference in degree or speed of learning between elevations. This suggests a consistent benefit of social learning in great tits across environments rather than the increased benefit with elevation that we predicted. Our study showed that although many individuals exhibited the use of local enhancement, only a few attempted to copy the demonstrator’s action, which suggests that more advanced social learning (e.g. action copying) may require more demonstration, possibly combined with asocial trial and error learning. Finally, we found consistent individual differences in the use of local enhancement but not action copying suggesting some individuals are more prone to using social information for finding food patches than others irrespective of environmental conditions. These results improve our understanding of how the external environment impacts social learning and furthers the debate on the role that asocial trial and error learning plays in social learning (see below).

Social learning should be more advantageous in harsh environments where the consequences of missed foraging opportunities are more severe than in benign environments. Although evidence for social learning was clear, we found no difference in either degree or latency of local enhancement and action copying between elevations. One possible explanation for why great tits did not differ in social learning between elevations is that social learning might be equally advantageous at high and low elevations but compensate for different foraging challenges at either elevation. For example, if food patches at high elevation are small and ephemeral, using social information may only provide small benefits in the same order as at low elevation where patches might be more common but competition is higher. Alternatively, although learning is a form of plasticity as it allows for flexible responses to changes in the environment (Boyd and Richerson 1988; Dukas 2004), the use of social learning could also be plastic. If so, our approach of housing individuals from high and low elevation under standard conditions for several days before testing might have led to a convergence in social learning use among birds from different elevations. The contrast between patterns in a common garden and studies in the wild would help us understand plasticity in social learning. Indeed, despite the initial discussion of plasticity in cognition two decades ago (Dukas 2004), it remains relatively unexplored to date (Cauchoix et al. 2020).

Differences across species with specialists versus generalists foraging strategies may also generate species differences in the use of social learning under different environmental contexts (Klopfer 1961; Sasvári 1979; Lefebvre and Giraldeau 1996; Galef and Giraldeau 2001). However, empirical results to date do not necessarily reveal a clear pattern. For example, great tits are extreme habitat and diet generalists (Fisher and Hinde 1949; Betts 1955; Estók et al. 2010) that learn socially (Sasvári 1979; Brodin and Urhan 2014; Aplin et al. 2015; Brodin and Urhan 2015) and do so regardless of environmental harshness in our experiments. In contrast, mountain chickadees which are food-caching specialists show reduced use of social learning in harsh, high-elevation populations compared to conspecifics at low elevation, possibly because asocial learning of cached food is more valuable at higher elevations (Heinen et al. 2021, 2022). However, this pattern does not hold for all food-caching specialists. Black-capped chickadees cache food in winter and rely on social learning more in harsh rural areas compared to urban areas where supplemental feeding generates stable food patches (Jones et al. 2017). Other Paridae species, including both specialist food-caching marsh tits (*Poecile palustris*) and generalist blue tits, show poor social learning compared to great tits (Sasvári 1979; Urhan et al. 2017). Clearly, the relationship between foraging strategy and social learning that has generally been assumed (Lefebvre and Giraldeau 1996; Galef and Giraldeau 2001) is not so straightforward when factoring in environmental harshness. Direct measures of the impact of “environmental harshness” on the organisms (Love and Wagner 2023; Makuya et al. 2023) and the size, stability, and ease of finding new foraging patches which impact the value of socially vs asocially acquired information would greatly improve our understanding of how species ecology and the environment shape social learning use.

Most, if not all, studies of social learning inadvertently include asocial learning as it is very difficult to separate the effects of copying a tutor from an individual’s own experience during the learning process. In our study, we aimed to separate social from asocial learning by only testing observers up until the first trial in which they sampled an item to prevent asocial trial-and-error learning (Galef 1995). Furthermore, we did not provide mealworms behind correct answers during social learning testing of the observer to limit their use of alternative cues (e.g. sound or smell). Finally, we removed the demonstrator, so it was not present during observer testing to separate social learning outcomes from a social facilitation of asocial learning (Klopfer 1961; Shettleworth 2010). This approach provided mixed results. Although we found evidence that the presence of a demonstrator increased the probability of approaching and interacting with the foraging task, individuals did not copy the correct choice more than expected by chance. This result suggests that individuals may require more demonstrations and that behaviour initially learnt by social learning requires fine-tuning via asocial learning or, most likely, both. Many studies that provide evidence of social learning have continuous contact with tutors and do not limit reinforcement by asocial trial and error (Terkel 1995; Marchetti and Drent 2000; Thornton and McAuliffe 2006; Mesoudi 2011; Guenther and Brust 2017). For example, Aplin et al. (2015) followed the spread of seeded solutions to challenging foraging tasks in flocks of great tits to show the establishment of traditions via social conformity. These studies show that social learning occurs, but do not quantify the possible contribution of asocial learning to social learning. Indeed, in Aplin et al.’s study, social learning clearly instigates the behavioural tradition in each flock, but reinforcement by asocial learning is likely a key force after the initial observation. More generally, learning socially might simply act as a dynamic source of information that introduces behavioural variation to be fine-tuned based on the personal experience later (Galef 1995), yet separating these effects remains a key challenge.

Social learning can occur to different degrees, from local enhancement to copying complex task solutions, reflecting an increase in required cognitive engagement (Hoppitt and Laland 2008). Indeed, we found that only a few individuals who used local enhancement, generally considered a simple form of social learning, also used action copying which involves a more complex cognitive process (Zentall 2006; Hoppitt and Laland 2008). A possible explanation of this may be that the time and energy investment required for copying the actions needed to accurately solve the task is unsustainably high compared to the combined use of local enhancement and asocial learning. Alternatively, it is possible that added costs associated with accurate action copying—either cognitive processing or additional observations—may mean that it is only profitable for very challenging tasks. Great tits forage in flocks where local enhancement should be favoured (Krebs et al. 1972), but forage opportunistically on many different food sources such that copying specific actions to get food may rarely be useful. Conversely, the accuracy of action copying might require multiple demonstrations and task performance with a demonstrator which was not part of our experimental design. Additional social learning opportunities and feedback via asocial learning may explain why others have found accurate action copying in great tits previously (Brodin and Urhan 2015; Hämäläinen et al. 2020), whereas we did not. If asocial learning is an integral component of action copying, providing such opportunities in future experiments could reveal differences in social learning among populations from different elevations.

If the propensity to learn socially is a personal information-gathering tactic, we might expect to find consistent variation among individuals in how they responded to task demonstration. Furthermore, we might expect that the propensity to use social information might differ among individuals from populations that differ in the value of social information as a result of harsh environmental contexts. Few studies have attempted to estimate the repeatability of social learning but those who do generally show moderate to high repeatability, *r* = 0.30–0.52 (Guenther and Brust 2017; Watson et al. 2018). Although we did not find an effect of the environment on the propensity to use social learning (Table 3), we did find significant repeatable among individual variation across tasks in local enhancement, but not for action copying. Furthermore, the degree of repeatability in social information use was not influenced by the elevation of origin of the bird (i.e. partial R of the environment as a moderator was not significant). This suggests some individuals consistently use social information to cue into and approach feeding patches more than others as expected for species that forage in groups. The lack of repeatability in action copying could be because there is little variation among individuals or because individuals are not consistent in whether they copy the actions of tutors. In the latter case, one possibility is that the two tasks we presented required different motor or cognitive skills such that one would not necessarily expect individuals to show similar behaviour across the two tasks. Alternatively, individuals may not show consistent action copying behaviour in general because it is not needed in great tits, as we argue above. Individual variation in local enhancement can be advantageous in group feeding contexts that include both producers (asocial learning users) and scroungers (social learning users) in a stable equilibrium or as mixed strategies (Barnard and Sibly 1981; Reichert et al. 2021). However, whether social learning of action copying follows producer–scrounger dynamics and to what degree it shift with population structure or ecology remains to be explored.

Studying cognition in an ecologically relevant context is a central tenant of cognitive ecology (Cauchoix and Chaine 2016; Morand-Ferron 2022), which allows comparisons among individuals, populations, and species to understand how cognition is shaped by the environment. Our findings that generalist great tits do not show an increase in the degree of social learning with increased environmental harshness contrasts with results from mountain chickadees which showed decreased social learning use with increased environmental harshness as expected from a caching species. We suggest that a fruitful framework to understand such differences could be by examining the costs and benefits of social learning given the species’ behavioural strategies and population ecology (Morand-Ferron et al. 2016). The benefits of social learning are clear in a variety of taxa (Griffin 2004; Thornton and McAuliffe 2006; Manassa and McCormick 2013; White et al. 2017), but context-dependent costs have largely been ignored. Costs have been observed in other forms of cognition (Mery and Kawecki 2003; Cole and Quinn 2012), and it seems likely that social learning carries costs under some contexts (Giraldeau et al. 2002). The cost–benefit relation of social learning can change over time, including short time scales, but examinations of plasticity in social learning use are lacking. Regardless, if the costs and benefits of social learning are context-dependent, then manipulations of contexts could be fruitfully used to measure these costs and benefits. Such an approach could provide important insights into the role that ecology and behavioural strategies play in the evolution of social learning.

## Supplementary Information

Below is the link to the electronic supplementary material.

Supplementary file1 (DOC 122 KB)

## Data Availability

Data and code used in the project are available in the Figshare Digital Repository: https://figshare.com/s/b280a6d32110e63c9087 (Isaksson et al. 2022).
